# Enhancement of Spurious Signal Suppression in Microstrip Mixers by Load Resistor Termination

**DOI:** 10.3390/s25020355

**Published:** 2025-01-09

**Authors:** Catur Apriono, Abdurrasyid Ruhiyat, Farohaji Kurniawan, Arokiaswami Alphones, Fitri Yuli Zulkifli

**Affiliations:** 1 Department of Electrical Engineering, Universitas Indonesia, Depok 16424, Indonesia; abdurrasyid.ruhiyat@ui.ac.id (A.R.); yuli@eng.ui.ac.id (F.Y.Z.); 2 Research Center for Aeronautics Technology, National Research and Innovation Agency, Jakarta Pusat 10340, Indonesia; faro001@brin.go.id; 3 School of Electrical and Electronic Engineering, Nanyang Technological University, 50 Nanyang Avenue, Singapore 639798, Singapore; ealphones@ntu.edu.sg

**Keywords:** microstrip, mixer, reflection, spurious, load resistor

## Abstract

The mixer is an essential component in RF transceiver subsystems. It has a role in shifting the signal frequency for more convenient processing of up-conversion as well as down-conversion. Despite generating the desired signal, the mixer also generates spurious noise that wastes power and reduces the performance of the overall system. This research proposes the design of a microstrip mixer that can enhance the suppression of spurious signals by utilizing the characteristics of the load resistor termination. The simulation results show that by adding the load resistor, the parasitic electrical field on the diode and the reflection signal inside the mixer can be reduced. The measurement result also validates that the suppression of the spurious signal is enhanced, where the signal-to-noise ratio can reach above 50 dB on the single-balanced mixer and above 60 dB on the double-balanced mixer.

## 1. Introduction

**Figure 1:**
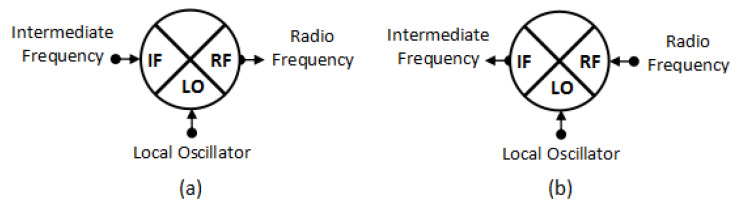
(**a**) Up-conversion and (**b**) down-conversion.

The mixer is an essential component of an RF transceiver subsystem. It is still being researched today and widely used for various applications [1,2,3,4,5,6]. The main function of the mixer is to translate or shift the signal frequency while maintaining its properties [7]. On the transmitter side (up-conversion), a signal that has a lower frequency (IF) will be translated to a higher frequency (RF). Transmitting signals with higher frequencies or shorter wavelengths is more convenient because the size of the component that processes the signal will be smaller. On the receiver (down-conversion), the mixer will translate the signal that has a higher frequency (RF) to a lower frequency (IF), which is more convenient for signal processing [8]. These two processes are shown in Figure 1.

The passive mixer uses nonlinear components such as a diode for the mixing process [9]. The diode will behave as a switch and is controlled by the polarization of the LO (Local Oscillator) signal. The LO signal turns the diode into condition on (short) and off (open) [8]. In this process, especially when the LO signal is relatively high, an electrical field will be formed between diode pins (charged). This electrical field needs to be discharged before the diode enters the off-state (open) condition. The additional time delay for the discharging process makes the on-state of the diode much longer than the off-state. Furthermore, it also has impacts on the undesired conversion loss and isolation values [10].

The parasitic electrical field on the diode can be removed by providing a DC ground path without disturbing the RF and IF signals. The common choice is to use short stubs or inductors [11]. The short stub acts as a band-pass filter that passes through the interested signal but will act as a short circuit for the DC component signal. The grounded inductor also has similar properties that will block high-frequency signals and bypass the DC signal. Nevertheless, in addition to the electrical field (charging), there remains a problem regarding the reflection inside the mixer. Based on simulation and measurement, the reflection inside the mixer will degrade the performance of the mixer by producing unwanted signals known as spurious signals.

This paper presents a design of a microstrip mixer that can remove the parasitic electrical field, reduce the reflection, and enhance the suppression of spurious signals. It utilizes the characteristics of load resistors to absorb the reflection signal inside the mixer and, at the same time, become a DC ground path to discharge the accumulation of electrical field on the diode [11]. The measurement results show that the suppression performance of the mixer is increased to above 50 dB on a single-balanced mixer and to above 60 dB on a double-balanced mixer.

In general, this paper starts with a brief explanation of the microstrip mixer’s basic design. The next section presents the simulation and measurement results of the conventional microstrip single-balanced mixer. The results will be the reference for any further modifications. The following section presents the results of the modified single-balanced mixer equipped with short stubs and inductors, which are existing methods used to remove the electrical field from diodes. The next section presents the results of the proposed mixer design equipped with load resistors. The behavior of the simulated mixer is observed to see its impact on the spectrum of the produced signal.

## 2. Basic Design

### 2.1. Conventional Mixer

**Figure 2:**
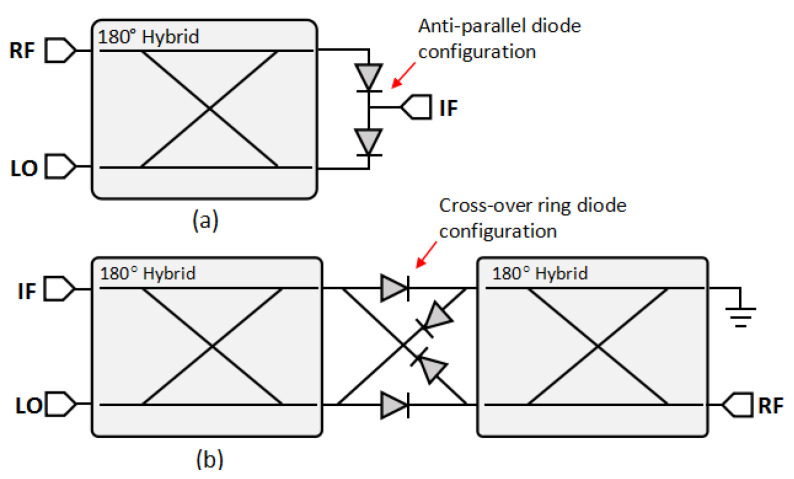
The configuration of (**a**) single-balanced (SBM) and (**b**) double-balanced mixer (DBM).

Figure 2 shows the conventional configuration of single-balanced (SBM) and double-balanced (DBM) mixers. These two configurations are commonly used in RF subsystems because they can compromise complicated design and performance. The single-balanced mixer uses an anti-phase (180°) hybrid coupler to drive the diode, which has an anti-parallel configuration. The double-balanced mixer uses two anti-phase hybrid couplers and is connected by four diodes with a cross-over configuration [11]. The SBM uses a diode Infineon BAT15-04W series [12], and the DBM uses a diode Infineon BAT15-099R series [13]; both diodes are built in an integrated circuit (IC) package.

### 2.2. Modified Mixer

**Figure 3:**
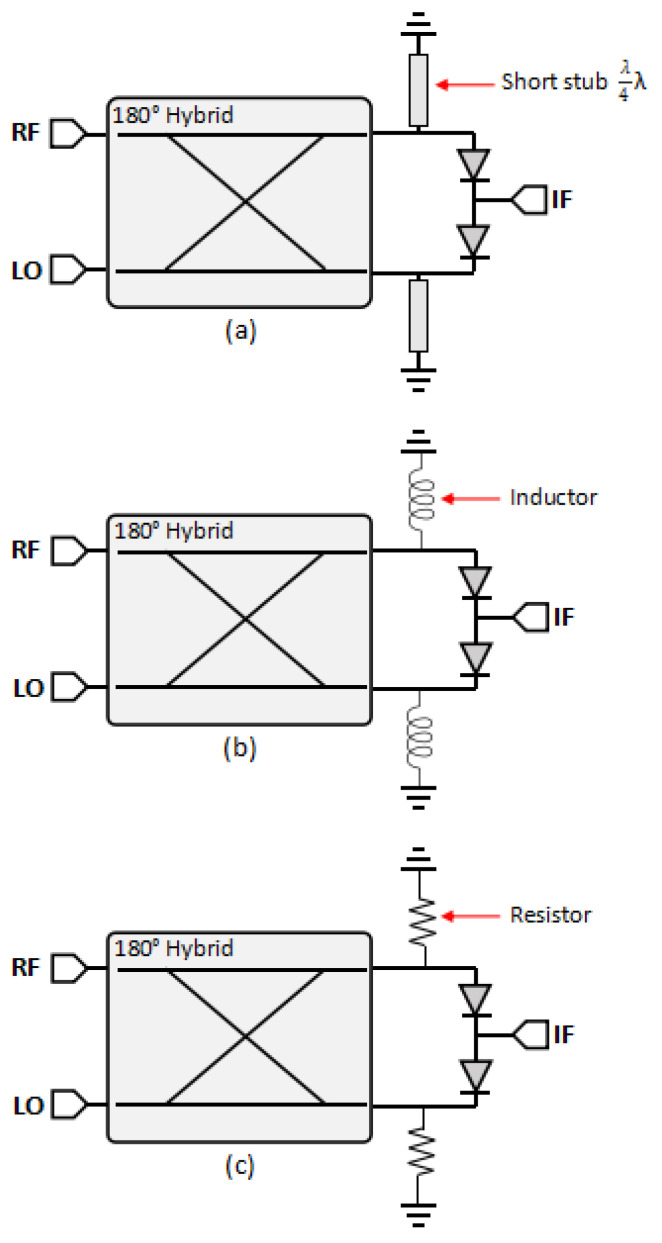
Configuration of (**a**) SBM with short stubs, (**b**) SBM with inductors, and (**c**) SBM with load resistors.

Figure 3 shows the modified single-balanced mixer equipped with short stubs, inductors, and resistors. Short stubs and inductors are established solutions to prevent the diode from forming a parasitic electrical field (charging) by providing a DC ground path [7]. Meanwhile, the resistor is the proposed design to not only remove the charging area but also absorb the reflection signal inside the mixer.

The basic and modified mixers are simulated, fabricated, and measured to confirm their impact on the output signal spectrum. The observation is focused on the mixer at the transmitter side (up-conversion) and port 3 as the source of the signal (LO) for driving the diodes. The simulation is carried out using CST microwave studio, and the measurement is using the N9917A FieldFox handheld microwave analyzers from Keysight.

### 2.3. Dimension and Material

**Figure 4:**
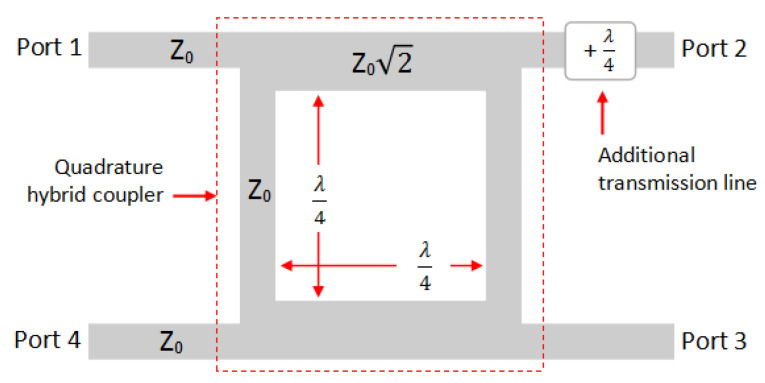
Configuration of anti-phase (180°) hybrid coupler.

As shown in Figure 2 and Figure 3, the mixer is composed of an anti-phase (180°) hybrid coupler. In this research, the coupler is constructed by a quadrature (90°) hybrid coupler with modification on one of the output ports. A transmission line that has a length of quarter wave is added to transform the quadrature coupler into an anti-phase coupler, as shown in Figure 4. This design is preferred because easier to integrate and can avoid the cross-transmission line (interference) compared to the conventional anti-phase rat-race coupler [14]. The two output ports are then connected to the diode to form a single-balanced mixer.

The mixer is fabricated by using substrate RT/duroid 5880, which has a permittivity ($\epsilon_{r}$) value of 2.2, substrate thickness (*h*) of 0.51 mm, a dissipation factor (tanδ) of 0.0009, and conductor thickness (*t*) of 0.035 mm. The substrate that has a higher permittivity and low thickness is preferable because it can lead to a narrower transmission line [11].

The mixer is intended to be used on a synthetic aperture radar (SAR) system, which has an LO fixed frequency of 5.5 GHz and a maximum chirp frequency of 75 MHz. The width (w) and length of the coupler transmission lines were then obtained by using Equations (1)–(3) and are summarized in Table 1. Equation (1) is used to obtain the effective permittivity value ($\epsilon_{e}$), Equation (2) is used to obtain the impedance value (*Z*), and Equation (3) is used to obtain the transmission line length, where *c* is the speed of light [11].

(1) $$\epsilon_{e}=\frac{\epsilon_{r}+1}{2}+\frac{\epsilon_{r}-1}{2}{\left(1+\frac{12h}{w}\right)}^{-0.5}-0.217\left(\epsilon_{r}-1\right)\frac{t}{\sqrt{wh}}$$

(2) $$Z=\frac{120\pi \epsilon_{e}^{-0.5}}{\frac{w}{h}+1.393+0.0667\ln\left(1.444+\frac{w}{h}\right)}$$

(3) $$\lambda =\frac{c}{f\sqrt{\epsilon_{e}}}$$

Adjustments to the length of the transmission line on the coupler are necessary to achieve an optimal response at a frequency of 5.5 GHz while the width remains unchanged to maintain the impedance value. Due to design discontinuities, such as curved corners or transmission line intersections, the electrical length of the transmission line may differ slightly from the calculated value [9]. The final dimensions were determined through continuous simulation and are presented in each section.

## 3. Simulation and Measurement

### 3.1. Conventional Mixer

**Figure 5:**
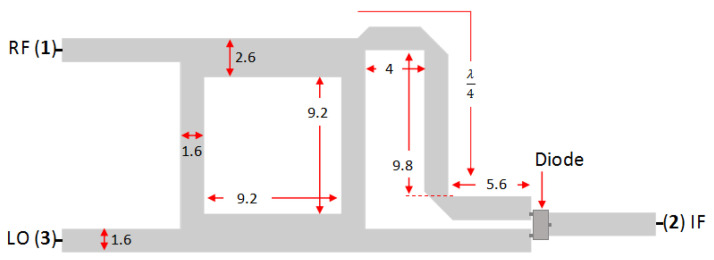
Dimension and configuration of basic SBM (dimensions are in mm).

Figure 5 shows the dimensions and configuration of the conventional (basic) microstrip single-balanced mixer. The RF and LO ports are on the same side, and the IF port is on the opposite side [8] connected by two diodes with anti-parallel configuration in the form of an integrated circuit (IC) package.

**Figure 6:**
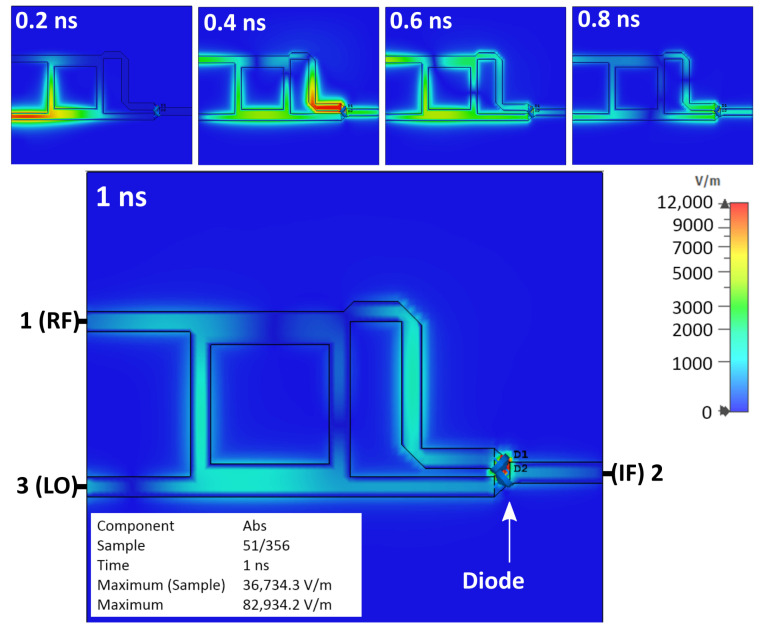
Simulation results of basic SBM.

Figure 6 shows the simulation results of the basic single-balanced mixer, which are the sequence of the observed electrical field behaviors at an interval time of 0.2 nanoseconds (ns). It shows that the diode is charged, and an electrical field is formed, represented by the red area around the diode. The reflection in the mixer was also noticed and remained for a few nanoseconds.

**Figure 7:**
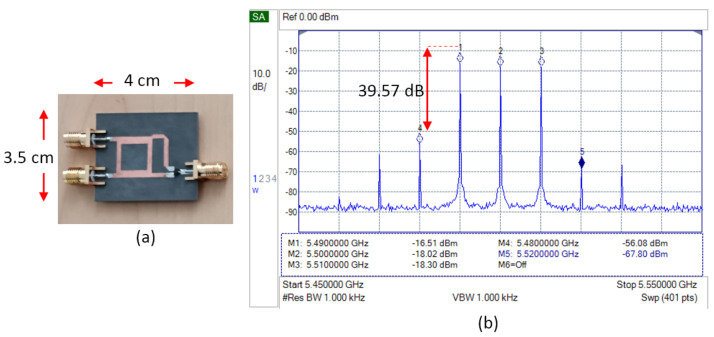
(**a**) Fabrication and (**b**) spectrum measurement result of basic SBM.

Figure 7 shows the fabrication and measurement results of the basic single-balanced mixer. The measurement is focused on the spectrum signal on the transmitter side. The frequency of the LO signal is 5.5 GHz, and the IF signal sample is 10 MHz. At the output, two desired signals appear, which have a frequency of 5.51 GHz at the upper sideband (USB) and 5.49 GHz at the lower sideband (LSB) [15]. The mixer also produces LO leakage and spurious signals, which have the highest power at frequencies 5.48 GHz (spurious LO-2IF) and 5.52 GHz (spurious LO+2IF). These results will be a basic reference to be compared with any further modification.

### 3.2. Modified Mixer (Short Stub)

**Figure 8:**
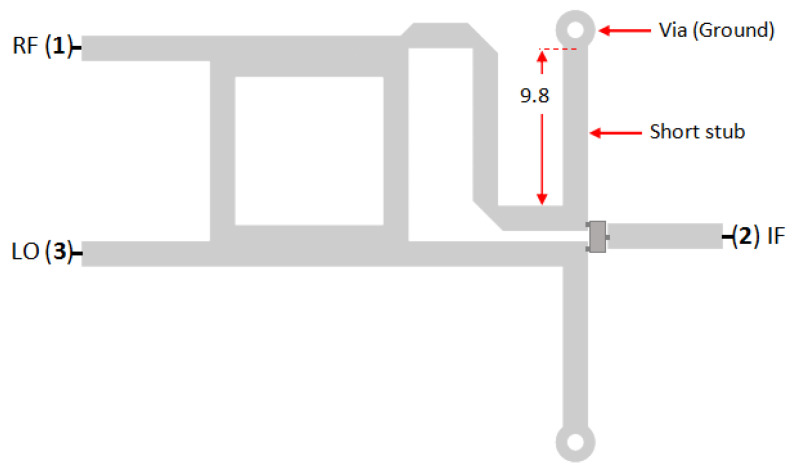
Dimension of SBM with short stub (dimensions are in mm).

Figure 8 shows the dimensions and configuration of a single-balanced mixer equipped with short stubs. It has a quarter length of the LO signal and is connected to the ground by using a via hole. The short stub acts as a band-pass filter [8], passing the interested signal (LO and RF), and becomes a DC ground path to discharge the accumulated electrical field on the diode.

**Figure 9:**
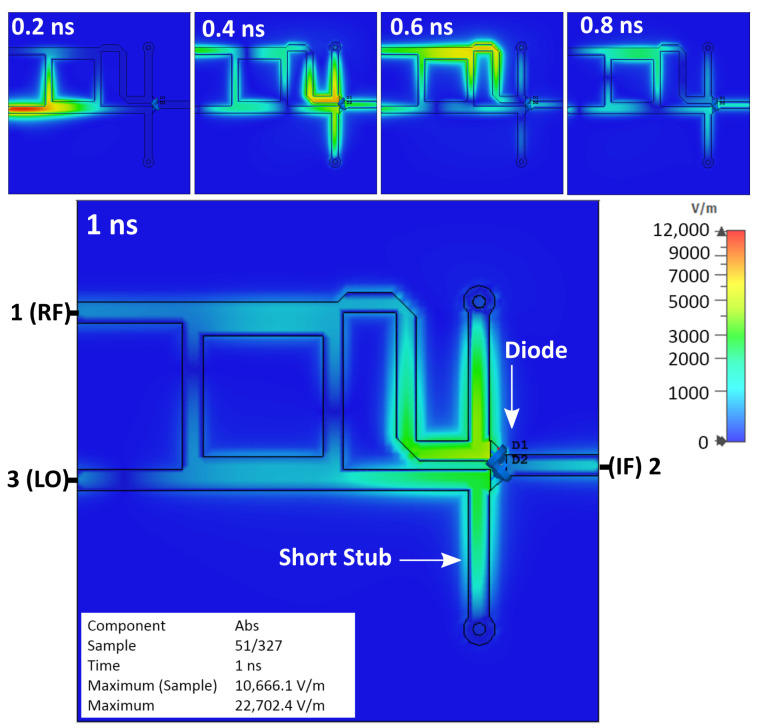
Simulation result of SBM with short stubs.

Figure 9 shows the simulation result of the single-balanced mixer, which is equipped with short stubs. The area around the diode has a blue color that can be interpreted as a low electrical field. This means that applying the short stubs can prevent the accumulation of electrical field on the diode. Nevertheless, the reflection inside the mixer remains.

**Figure 10:**
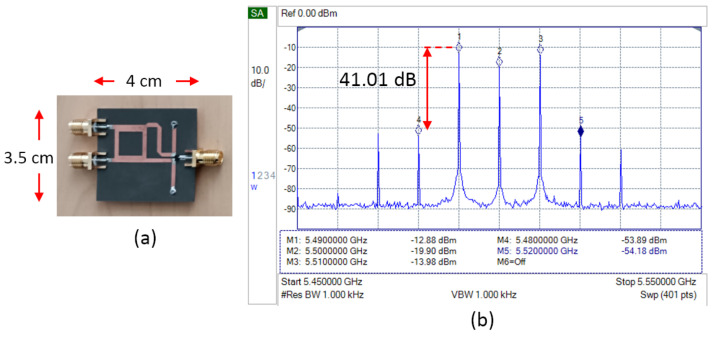
(**a**) Fabrication and (**b**) spectrum measurement result of SBM with short stub.

Figure 10 shows the measurement results of a single-balanced mixer equipped with two short stubs. Compared to the basic configuration of SBM, it improves the output power (LSB and USB) and the LO leakage suppression. The LSB increased by 3.63 dB, the USB increased by 6.43 dB, and the LO leakage decreased by 1.88 dB. The spurious signal suppression also increased by 1.44 dB.

### 3.3. Modified Mixer (Inductor)

**Figure 11:**
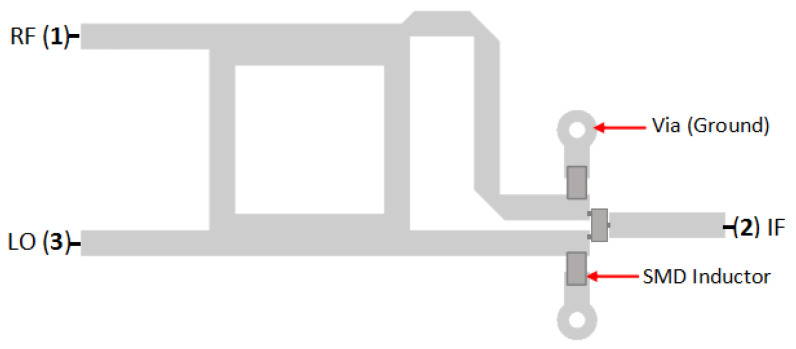
Configuration of SBM with inductor.

Figure 11 shows the configuration of a single-balanced mixer equipped with two surface-mount device (SMD) inductors. The inductor acts as an RF choke (RFC) that will block high-frequency signals and become the passage for the DC signal. To achieve this purpose, the inductor must have a high impedance value of at least ten times the interested signal [9,16,17].

The value of the inductor is obtained through Equation (4), where *f* is the frequency of the LO signal (5.5 GHz), *L* is the inductance value of the inductor, and $X_{L}$ is the impedance value of the inductor. With the desired impedance at 500 Ohm, the inductance value is obtained at 14.5 nH. Considering the availability of the component on the market, the inductor value is replaced with 18 nH, which has an impedance value of 622 Ohm.

(4) $$L=\frac{X_{L}}{2\pi f}$$

**Figure 12:**
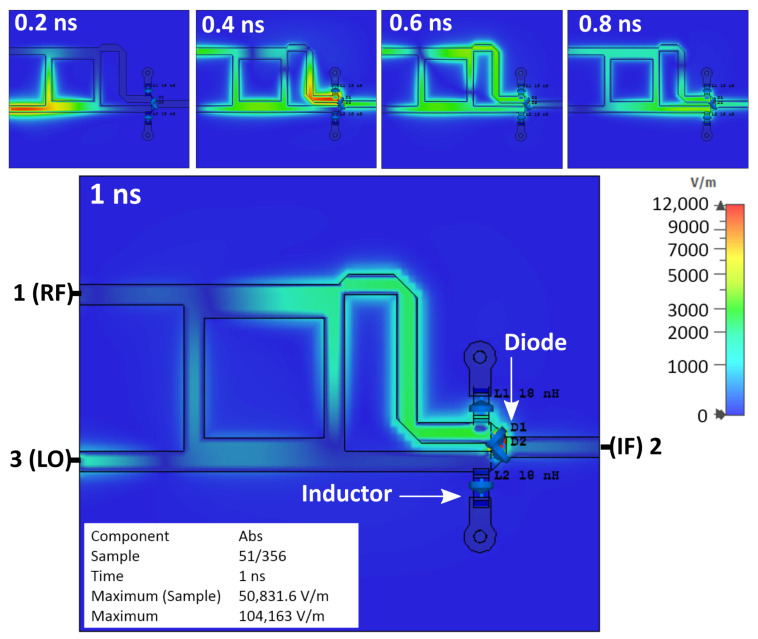
Simulation result of SBM with inductors.

Figure 12 shows the simulation result of a single-balanced mixer equipped with two inductors. The electrical field that was expected to be discharged remained on the diode. The area around the diode has a red color, indicating that the electrical field is formed. The reflection signal inside the mixer can also be noticed. Based on the simulation, applying the inductor does not discharge the parasitic electrical field on the diode or remove the reflection inside the mixer.

**Figure 13:**
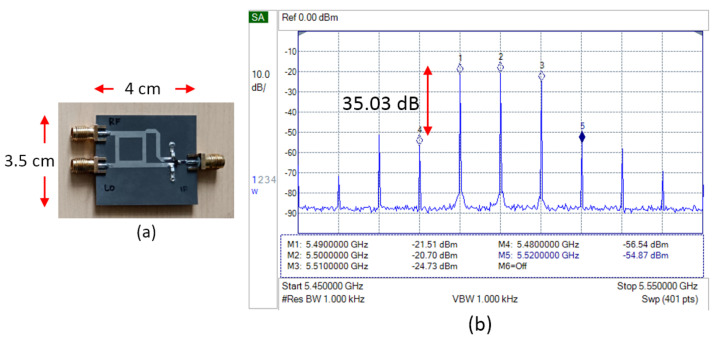
(**a**) Fabrication and (**b**) spectrum measurement result of SBM with inductor.

Figure 13 shows the fabrication and measurement results of the single-balanced mixer equipped with inductors. The signal power of the desired signal (LSB and USB), LO leakage, and suppression of spurious signals decreased. There is no noticeable improvement, only the reduction in power on the produced signals.

### 3.4. Modified Mixer (Load Resistor)

Based on previous simulations and measurements, the mixer has two problems: the electrical field on the diode (charged) and signal reflection inside the mixer. Both cases can be observed through the simulation. The red area shows the electrical field around the diode, and the reflection is shown by the signal that travels in the opposing direction from the incoming signal after it reaches the diode.

Applying the inductor to the mixer does not show a noticeable improvement. Meanwhile, the short stub can remove the parasitic electrical field and increase the quality of the output signal. Nevertheless, the reflection signal inside the mixer remains. Therefore, the implementation of load resistors is proposed to solve these problems.

**Figure 14:**
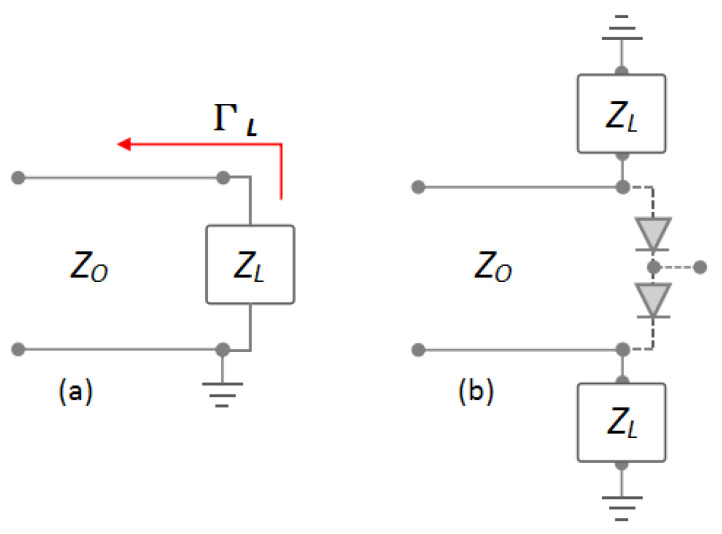
Illustration of (**a**) the reflection coefficient and (**b**) the application of load resistor on the mixer.

Figure 14 shows the illustration of the reflection coefficient (Γ) and the application of the load resistor on the mixer. Based on Equation (5), the reflection coefficient will have a 0 (zero) value while $Z_{O}$ is equal to $Z_{L}$ [11]. In this condition, the maximum power transfer will occur, and no reflection signal will appear.

In reality, maintaining matching impedance over the wideband spectrum is not easy. At least, the impedance value of the load resistor must be as close as possible to the impedance of the transmission line to get optimum suppression of the reflection signal. Therefore, the resistor with a value of 50 Ω will be used as a load resistor.

(5) $$\Gamma_{L}=\frac{Z_{L}-Z_{O}}{Z_{L}+Z_{O}}$$

**Figure 15:**
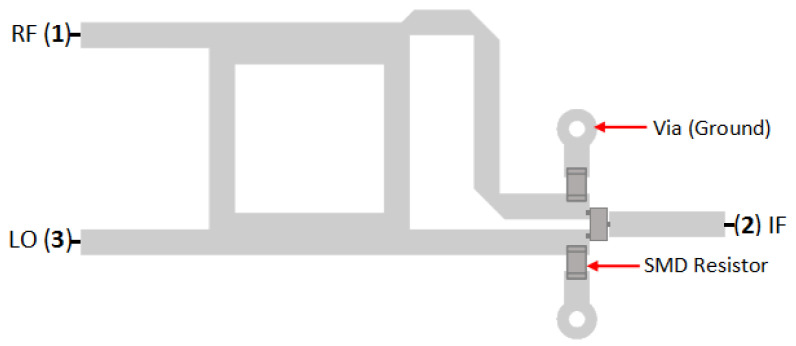
Configuration of SBM with load resistor.

Figure 15 shows the load resistor placement at the end of the transmission line of the coupler, next to the diode. It is expected to absorb the main signal and the reflection inside the mixer, similar to the function of the resistor as termination or dummy load [18]. This resistor will also become a DC ground path to prevent (discharge) the accumulation of electrical field on the diode.

**Figure 16:**
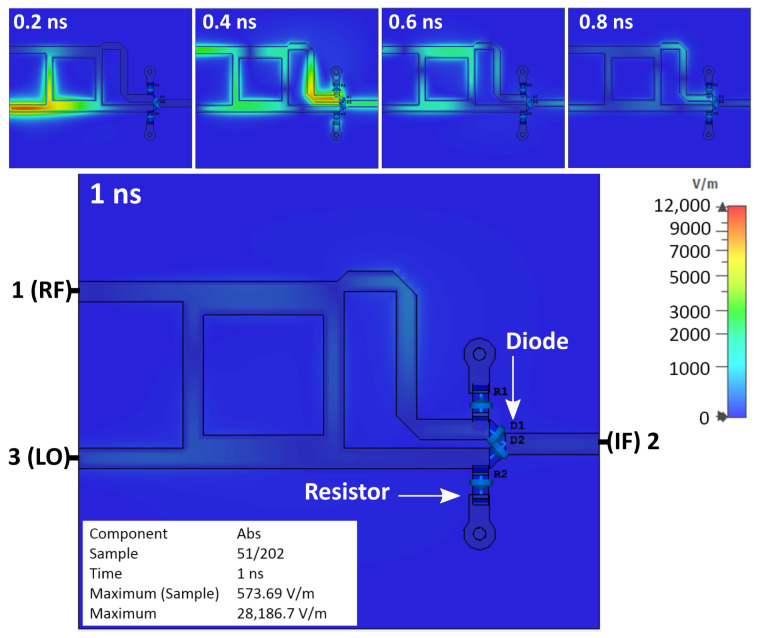
Simulation results of SBM with load resistor.

Figure 16 shows the simulation result of a single-balanced mixer equipped with load resistors. The area around the diode is blue, which means it has a low electrical field value. The reflection signal inside the mixer reduces faster compared to previous configurations.

**Figure 17:**
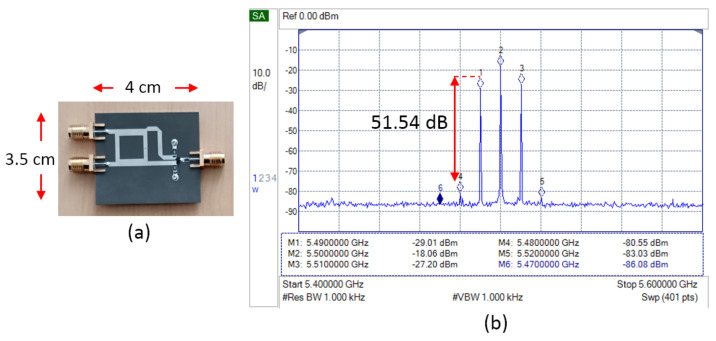
(**a**) Fabrication and (**b**) spectrum measurement result of SBM with load resistor.

Figure 17 shows the fabrication and measurement result of a single-balanced mixer with two load resistors. The LSB and USB signal is reduced below the LO leakage, but the spurious signal is further reduced, reaching a signal-to-noise ratio of 51.54 dB. Because of the promising result, the load resistor was then applied to the double-balanced mixer.

The double-balanced mixer cannot be performed in simulation because the diode has a cross-over ring configuration. The two diodes occupy the same area mesh, which leads to errors during simulation. Therefore, the load resistors are implemented directly into the fabricated mixer.

**Figure 18:**
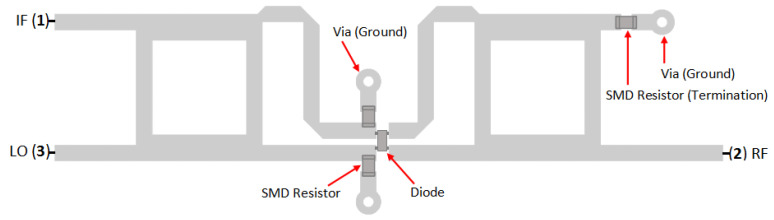
Configuration of DBM with load resistors.

Figure 18 shows the load resistor implementation on the double-balanced mixer. It is composed of two anti-phase hybrid couplers and connected by four diodes in a cross-over ring configuration. The LO and IF ports are on the same side, and the RF port is on the opposite side. The load resistor is placed on the LO port side, next to the diode. The unused port is terminated by a 50 Ω SMD resistor [14].

**Figure 19:**
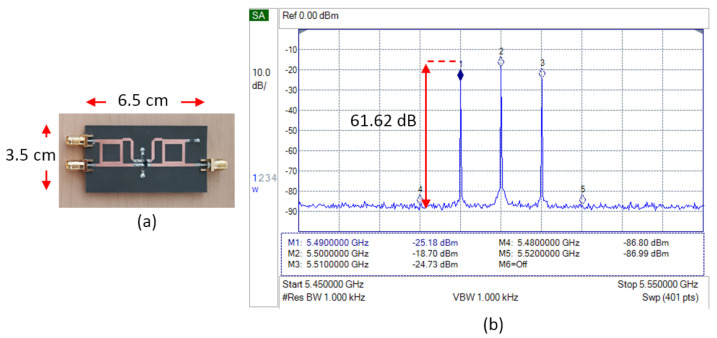
(**a**) Fabrication and (**b**) spectrum measurement result of DBM with load resistor.

Figure 19 shows the measurement results of the modified double-balanced mixer. The significant improvement in spurious signal suppression can be clearly seen. The spurious signal cannot be further measured because it is expected to be below the noise floor or outside the dynamic range of the spectrum analyzer. The distance between the output signal (LSB) to the noise floor (SNR) reaches 61.62 dB.

Due to the limited measurement equipment, which was a single signal generator for the LO and an FPGA (Field Programmable Gate Array) to generate the IF signal, this research has not considered the third-order intermodulation (IM3) and third-order intercept point (IP3). To measure these values, two signal generators are required to provide the fundamental signals, f1 and f2, and one signal generator to provide the LO signal.

## 4. Discussion

Table 2 summarizes the measurement results of the fabricated mixer. It shows that the implementation of the short stub on the mixer increases the LSB and USB power, reduces the LO leakage, and slightly increases the signal-to-noise (SNR). This performance is better than the conventional (basic) mixer.

Applying the inductor, which has a value of 18 nH on the mixer, does not improve the output signal quality. The noticeable change is the reduction in the LSB, USB, and LO leakage power. The SNR also decreased compared to the conventional mixer. These measurement results agree with the simulation, where the parasitic electrical field and the reflection still occur.

The implementation of load resistors on the single-balanced mixer reduces the LSB and USB signal power. The spurious signal power also experiences reduction, but much higher, which can increase the signal-to-noise ratio to above 50 dB. This result agrees with the simulation where the electrical field on the diode and the reflection inside the mixer can be removed faster, which leads to a higher reduction in the spurious signal.

The double-balanced mixer, which is equipped with load resistors, produced a better result compared to the implementation in the single-balanced mixer. As shown in Figure 19, the performance of the mixer is enhanced. The LSB and USB power is increased, and the spurious signal is estimated to be suppressed beyond the dynamic range capability of the spectrum analyzer and cannot be further measured. This configuration can achieve a signal-to-noise ratio above 60 dB.

**Figure 20:**
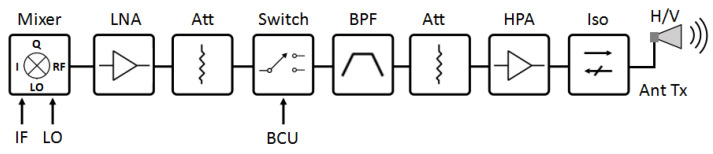
Schematic diagram of the RF SAR transmitter.

The modified double-balanced mixer has the highest spurious signal suppression. Even though the desired signal (LSB and USB) slightly decreased compared to the conventional single-balanced mixer, it can be compensated by an amplifier, as shown in Figure 20. A low-noise amplifier (LNA) will amplify the output signal from the mixer to obtain a suitable power level for further processing, followed by the adjustment of the attenuator to ensure the signal power remains safe for all other components. The signal and noise (spurious) will experience the same amplification. Therefore, a high SNR value is preferred rather than a high peak power signal because after amplification, the noise will remain low, and the desired signal can be seen clearly.

Since the systems use a direct conversion method to mix the IF and LO, the LO leakage will appear in the middle of the RF signal, between the lower sideband and the upper sideband. This research focuses on the discussion of the suppression of spurious signals because it affects the system performance in the wide bandwidth. The LO leakage will remain the same even if the bandwidth of the signal IF increases, different from the spurious signal, which will increase when the bandwidth of IF increases.

## Figures and Tables

**Table 1:** Width and length of the coupler transmission line.

Impedance	Width (w)	Length ($\frac{1}{4}\lambda$)
$Z_{0}$ = 50 Ω	1.6 mm	10.4 mm
$Z_{0}$$\sqrt{2}$ = 70.71 Ω	2.6 mm	10.2 mm

**Table 2:** Measurement result of fabricated mixer.

Configuration	Spurious LO-2IF 5.48 GHz (dBm)	Lower Side Band 5.49 GHz (dBm)	LO Leakage 5.5 GHz (dBm)	Upper Side Band 5.51 GHZ (dBm)	Spurious LO+2IF 5.52 GHz (dBm)
SBM (basic)	−56.08	−16.51	−18.02	−18.30	−67.80
SBM + short stub	−53.89	−12.88	−19.90	−13.98	−54.18
SBM + inductor	−56.54	−21.51	−20.70	−24.73	−54.87
SBM + resistor	−80.55	−29.01	−18.06	−27.20	−83.03
DBM + resistor	−86.80	−25.18	−18.70	−24.73	−86.99

## Data Availability

The original contributions presented in the study are included in the article. Further inquiries can be directed to the corresponding author(s).
